# Recent Advances and Implication of Bioengineered Nanomaterials in Cancer Theranostics

**DOI:** 10.3390/medicina57020091

**Published:** 2021-01-21

**Authors:** Ayushi Rai, Saba Noor, Syed Ishraque Ahmad, Mohamed F. Alajmi, Afzal Hussain, Hashim Abbas, Gulam Mustafa Hasan

**Affiliations:** 1 Department of Nanoscience, Central University of Gujarat, Sector 29, Gandhinagar 382030, India; adyashu@gmail.com; 2 Centre for Interdisciplinary Research in Basic Sciences, Jamia Millia Islamia, Jamia Nagar, New Delhi 110025, India; sabanooramu@gmail.com; 3 Department of Chemistry, Zakir Husain Delhi College, University of Delhi, New Delhi 110002, India; ishraque.ahmad@gmail.com; 4 Department of Pharmacognosy, College of Pharmacy, King Saud University, Riyadh 11451, Saudi Arabia; malajmii@ksu.edu.sa (M.F.A.); afzal.hussain.amu@gmail.com (A.H.); 5 Department of Medicine, Nottingham University Hospitals, NHS Trust, Nottingham NG7 2UH, UK; hashimabbas82@gmail.com; 6 Department of Biochemistry, College of Medicine, Prince Sattam Bin Abdulaziz University, P.O. Box 173, Al-Kharj 11942, Saudi Arabia

**Keywords:** cancer therapy, HPV-mediated cervical cancer, controlled drug release, anti-cancer efficacy, gold and silver nanoparticles, quantum dots, *Helicobacter pylori*-mediated gastric cancer

## Abstract

Cancer is one of the most common causes of death and affects millions of lives every year. In addition to non-infectious carcinogens, infectious agents contribute significantly to increased incidence of several cancers. Several therapeutic techniques have been used for the treatment of such cancers. Recently, nanotechnology has emerged to advance the diagnosis, imaging, and therapeutics of various cancer types. Nanomaterials have multiple advantages over other materials due to their small size and high surface area, which allow retention and controlled drug release to improve the anti-cancer property. Most cancer therapies have been known to damage healthy cells due to poor specificity, which can be avoided by using nanosized particles. Nanomaterials can be combined with various types of biomaterials to make it less toxic and improve its biocompatibility. Based on these properties, several nanomaterials have been developed which possess excellent anti-cancer efficacy potential and improved diagnosis. This review presents the latest update on novel nanomaterials used to improve the diagnostic and therapeutic of pathogen-associated and non-pathogenic cancers. We further highlighted mechanistic insights into their mode of action, improved features, and limitations.

## 1. Introduction

Cancer is a malignant disease involving abnormal cell growth due to the transformation of normal cells to tumor cells in a multistage pathway progressing from pre-cancerous lesion to malignant state. The uncontrolled growth of cancer cells is mostly associated with the gene mutations that occur after birth and are only rarely inherited [1]. Many factors trigger gene mutations, including pollution, exposure to ultraviolet light, and many unknown. In addition, infectious pathogens have been directly or indirectly associated with cancer etiology. Pathogen-induced oncogenesis has been attributed to the overall global cancer burden [2]. Pathogens associated with cancer are grouped as direct or direct carcinogens. A critical section of the viral genome is integrated into host DNA, resulting in the expression of viral oncogenes disrupting cell-cycle checkpoints, apoptosis, causing indefinite proliferation and cell immortalization. However, indirect pathogens do not induce oncogene expression but lead to chronic inflammatory conditions due to persistent infection [3]. As cancer is a complex disease and its therapy is still challenging and hence considered a serious health concern [2]. 

Although a considerable investment has been made in materials, manpower, and financial resources, numerous challenges remain due to the complexity of current clinical treatment modalities in cancer management [3]. To address these challenges, three approaches involving targeted therapy, combination therapy, and diagnosis by imaging are undertaken. Drug targeting is one of the major difficulties encountered during drug delivery at the tumor site [4]. The identification of appropriate molecular targets is required for developing target therapies [5]. The key role of molecular targeting is to develop and design a drug that can effectively bind to the target. However, it has been observed that somebody parts lack sites where an anticancer drug can bind. Therefore, designing drugs that target these areas has remained a challenge [6].

In cancer, drug resistance is a well-known phenomenon in which cancer becomes tolerant to either chemotherapy drugs or newer targeted treatment [7]. Cancer cells are known to develop drug resistance due to various reasons, including epigenetic changes or genetic mutations, upregulated drug efflux, and many molecular or cellular mechanisms. Although many advances have been made in cancer treatment in the last few decades, drug resistance remains a major obstacle causing increased mortality rate [8].

Cancer treatments currently include radiation therapy, targeted therapy, immunotherapy, cytotoxic chemotherapy, endocrine therapy, and surgery [9]. Anticancer drugs used in classical chemotherapy kills cancer cells by damaging their DNA, causing severely high toxicity. Both radiotherapy and chemotherapy have side effects on healthy cells and poor specificities for cancer cells. Monotherapy is not an efficient method to eradicate the tumor, as it becomes resistant to a single therapy [10]. Alternative methods have been adopted where combined therapy is used, which consists of two or more therapies to conquer the flaws of monotherapy [11,12].

The field of nanomedicine includes creating nanoscale materials with such properties that can be used to enhance cancer treatment efficiency [13,14]. Nanosized materials can be used as carriers for a cancer drug, cancer detection, and drug targeting by imaging techniques and controlled drug release [15,16,17]. Properties of nanomaterials such as electrical, mechanical, optical, magnetic, and biological are being leveraged to contribute to the diagnostics and therapeutics of cancer management [18,19]. Implications of nanomaterials used in the diagnosis and therapy of various types of cancer are listed in Table 1. In this review, we aimed to discuss current progress made to exploit advanced features of nanomaterials in Oncology. The most common and leading cancers worldwide include lung (2.08 million cases), breast (2.09 million cases), prostate (1.28 million cases), and skin cancers (1.04 million cases). This review highlights the latest developments and advancements of nanotheranostic formulations for breast, lung, skin, and prostate-related cancers, focusing on clinical advantages. In the light of recent original research and review articles reporting metal, non-metal, or lipid NPs and combinations, the theranostic potential of various bioengineered NPs with mechanistic action are outlined.

## 2. Nanoformulations in Cancer Diagnosis and Treatment

**Figure 1:**
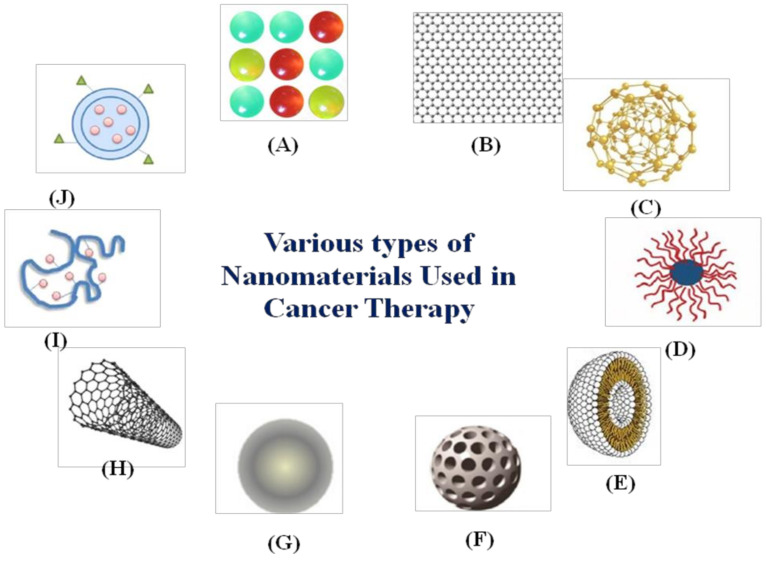
Various types of nanomaterials used in cancer therapy: (**A**) quantum dots, (**B**) graphene, (**C**) gold nanoparticles, (**D**) polymeric micelles, (**E**) liposomes, (**F**) silica nanoparticles, (**G**) magnetic nanoparticles, (**H**) carbon nanotubes, (**I**) polymer-drug conjugates, and (**J**) polymeric nanoparticles.

Nanomaterials are small-sized chemical substances with at least one dimension on a 1 to 100 nm scale with novel characteristics. In recent years, applications of nanotechnology have seen much research growth in the fields of material science, photonics, supramolecular assemblies, and drug delivery [40,41]. Nanomedicines are the result of the medical application of nanotechnology, promoting the development of nanoparticles of different kinds, for example, carbon nanotubes, liposomes, polymeric micelles, etc., as illustrated in Figure 1.

**Figure 2:**
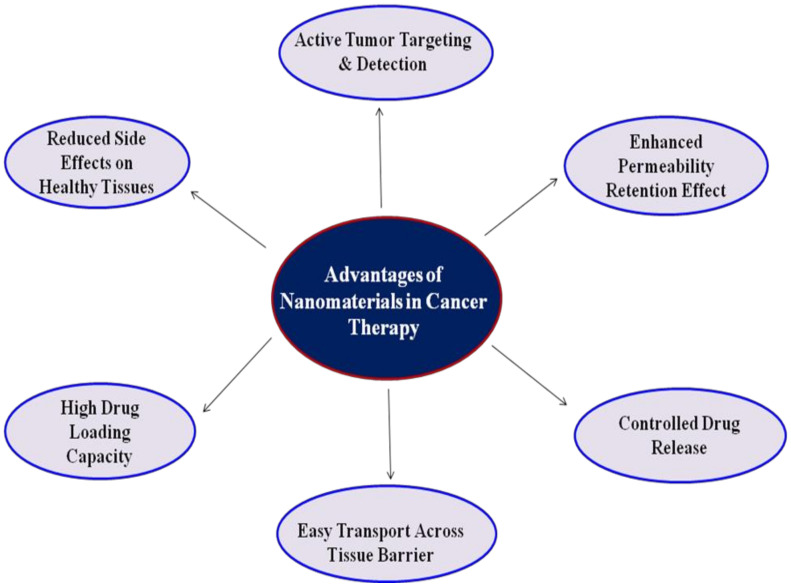
Advantages of nanomaterials in cancer therapy.

Great efforts have been employed to develop a different class of nanocarriers that can not only function as a cancer diagnostic agent but also inhibits therapeutic behavior [42]. Due to their small size, nanocarriers have higher permeability and retention, which shows more specificity and decreases the side effects of drugs, thus improving cancer treatment efficiency. Nanomaterials have a high surface to volume ratio, which is very useful to load a large amount of drug. Moreover, the high surface area of nanoparticles would allow co-loading of two or more types of therapeutic and diagnostic agents for synergistic therapy, such as photothermal therapy (PTT) and magnetic resonance imaging (MRI). This would efficiently improve therapeutic efficacy, and it will effectively avoid drug resistance due to monotherapy. In the last few decades, the use of nanomaterials in cancer therapy added some advantages and improved its therapeutic potency (Figure 2).

About 90% of cancers are non-infectious and caused by a combination of genetic and environmental factors. The accumulation of genetic changes in combination with other factors on the pathway to cancer increases with age. Exposure to harmful radiation, alcohol, tobacco, obesity, diet, and lifestyle are suspected risk factors for oncogenesis [43]. Here, we discuss the common non-pathogenic cancer types, including breast, lung, skin, prostate cancer, and nanomaterials’ implication in the diagnosis and treatment of common cancer types.

### 2.1. Breast Cancer

Breast cancer is one of the most common types of cancer and frequent in women. It occurs in mammary glands, ducts, or tissues of the breasts with one in eight women developing invasive breast malignancy [44]. Breast cancer is characterized by molecular alterations that can be used as diagnostic and prognostic markers. In addition to genetic factors (mutations in BRCA1 and BRCA2 genes), several non-genetic factors such as race, ethnicity, lobular neoplasia, radiation therapy, etc., contribute to the development of breast cancer [44]. Despite the scientific advancements in breast cancer diagnosis and treatment with conventional techniques, the increased mortality rate demands the implementation of novel therapeutics. Several classes of nanomaterials are widely used in the treatment of cancer, including surgery, hormone therapy, chemotherapy, radiotherapy, and immunotherapy. But these treatments have side effects and shortcomings that limit their efficacy [45]. For instance, radiotherapy causes damage to the surrounding tissues as it has poor selective efficacy. Prolonged use of chemotherapy can cause drug resistance [46]. Due to its anticancer efficacy, immunotherapy has attracted attention around the world, but it has failed to show the same effectiveness for all cancer patients [47]. Some important bioengineered nanomaterials of clinical value used in the diagnosis and treatment of breast cancer have been outlined below.

#### 2.1.1. Graphene Quantum Dots Conjugated with Herceptin and PEG

Graphene quantum dots (GQDs) are biocompatible and have photoluminescence properties that can be used to diagnose cancer at a low cost [48]. Herceptin (HCT), an antibody targeting HER2 receptors of breast cancer cells [49], upon conjugation with GQDs can differentiate between healthy and cancerous cells and have low toxicity and side effects. Another benefit of using GQDs on cancer diagnosis is improved targeting efficacy. Polyethylene glycol (PEG) can be attached to the GQDs to improve aqueous solubility and provide flexibility [50]. PEG provides stealth to the overall material to help evade the immune system, protects hydrophobic drugs in the core of the GQDs–HCT–PEG complex from degradation as well as rapid removal by renal filtration. Disulfide linkages can be used to improve the rapid release of the drug. Novel GSH-responsive degradable GQD-based nanoparticles (GQD-NPs) with encapsulated doxorubicin (DOX) were synthesized for breast cancer diagnosis and therapy [51]. It was noted that glutathione (GSH) in relevant concentrations caused the cleavage of disulfide linkages releasing DOX rapidly in a controlled manner.

#### 2.1.2. SWCNTs Functionalized with GNPOP for Targeted Diagnosis of Breast Cancer

Single-walled carbon nanotubes (SWCNTs) have unique structures, and hence, they are attractive nanomaterials for the development of biosensors [52]. CNTs have strong van der Waals interactions that hold them together firmly, forming bundles. Since SWCNTs are insoluble in all solvents, it is necessary to modify them to enhance their solubility by functionalization. Gold nanomaterials have huge potential in medicine and cancer therapy after functionating with different surface moieties [53]. Therefore, gold nano popcorns (GNPOP) with spheres that act as an electron reservoir and ends focusing the field on the apex can enhance Raman signals by several magnitudes. GNPOP has unique properties, as reported, [54] a novel hybrid nanomaterial for SERS probe that can be used for targeted diagnosis of breast cancer cells. The purpose of using this hybrid nanomaterial is that it can generate high temperature, which can improve the photothermal process by making it fast and effective, showing low toxicity and good biocompatibility. It was reported that the hybrid nanomaterial showed significant improvement in Raman signal intensity. The experimental data of HER2 negative MDA-MB breast cancer and HaCaT normal cell line explains that SERS assay is extremely sensitive to tumor cells, and can easily distinguish between other cell lines. The cancer cell detection and destruction took 20 min, and the photothermal response was far better than single nanomaterials.

#### 2.1.3. Hyd−AuNP−Apt Bioconjugate for the Diagnosis of Breast Cancer

An electrochemical method was developed for the simple detection of HER2 protein and HER2 overexpressing breast cancer cells by using a gold nanoparticle-based biconjugate [55]. Silver ions were used as reductant-attached bioconjugates for the proper deposition of silver on target molecules. The reductant was directly bonded to the electrode surface through the bioconjugate so that selective and controlled reduction of silver occurs. Hydrazine-attached bioconjugate for the direct detection of cancer cells and proteins has been used in this case [56]. The purpose of using hydrazine is to reduce silver ions to the silver metal, which is then conjugated to gold nanoparticles. A bioconjugate of hydrazine−AuNP−aptamer (Hyd−AuNP−Apt) was synthesized and reported [57]. The analysis of the silver deposited was done by square wave stripping voltammetry to determine the number of cancer cells. It is a novel report where breast cancer cells have been stained with silver selectively by interaction with Hyd−AuNP−Apt bioconjugate.

#### 2.1.4. Glutathione and Iron Redox Couple for Breast Cancer Ferroptosis Therapy

Ferroptosis is the accumulation of lethal lipid peroxide (LPO), an iron-dependent reaction that perturbs cellular redox homeostasis and potentially kills cancer cells. Fenton reaction between tumor cellular H_2_O_2_ and iron can generate reactive oxygen species leads to accumulation of LPO. However, in tumor regions, there is an insufficient amount of endogenous H_2_O_2_, and iron-mediated reaction cannot take place. Therefore, a Fenton-reaction independent LPO generator could effectively promote ferroptosis therapy where lipid peroxidation could occur in the absence of H_2_O_2_ by iron redox couple (Fe^2+/^Fe^3+^) [55]. Redox reaction between unsaturated lipids and oxygen requires electron transfer mediated by the coexisting of Fe^2+^ and Fe^3+^. Fe^3+^ can be reduced to Fe^2+^ by various reductive agents such as glutathione in tumor cells [56]. Recently, glutathione and iron redox couples were synthesized for Fenton reaction-independent ferroptosis therapy [21]. Here, a lipid peroxide generator that induces ferroptosis was used with a small particle size, which showed high tumor accumulation and permeability. Another benefit of using lipid peroxide generator was observed in in vivo and in vitro models, which indicated that it suppresses the tumor and does not show side effects.

#### 2.1.5. Biomimetic Lipid-Modified WS_2_ Nanosheets for Breast Cancer Therapy

Tungsten disulfide WS_2_ is a graphene-like layered material with a high drug loading capacity due to its large specific area [57]. WS_2_ nanosheet is beneficial for various reasons, such as the presence of many active sites on its surface that can be used for surface modification. It has low toxicity and a broad absorption band, which includes the near-infrared region, and therefore it can have applications in the field of optical therapy for cancer. PTT uses near-infrared light to produce local heat to kill cancer cells [58]. Easy operation and high efficiency are some of its advantages. To optimize tumor treatment, a combination of photothermal chemotherapy can be used by loading drugs on the nanosheets, which would improve therapeutic efficacy. Under physiological conditions, a few unavoidable shortcomings such as low biocompatibility, poor stability, and low cell uptake efficiency were observed. Cell membranes are components of living organisms ensuring stability in the intracellular environment and regulating control over the exchange of substances in and out of the cell. Cell membranes have excellent biocompatibility and, therefore, can be used to mimic the composition of biofilms. The modification of nanosheets with phospholipids can improve the stability and compatibility of inorganic materials. Therefore, biomimetic lipid-modified WS_2_ nanosheets were used as a substrate material to load doxorubicin for photothermal and chemo combination therapy [34]. The rate of drug loading and solution permeability after loading was seen to have greatly improved. Lipid modification increased the nano substrate’s stability, and the in vitro experiments showed that loaded drug exhibited pH-dependent controlled-release properties.

#### 2.1.6. Ce6-PTX@IR783 for Breast Cancer Imaging and Chemo-Sonodynamic Combination Therapy

Sonodynamic therapy (SDT) is an emerging cancer treatment as it has many advantages over photodynamic therapy (PDT), such as low phototoxicity and penetration depth of light [59]. It is a cost-effective, non-evasive, and easy to operate technique. It also provides therapeutic biosafety to normal tissue in the targeted area as the intensity and energy of the ultrasound used is quite low. Ultrasound sonosensitizer is necessary for SDT to enhance the therapeutic efficiency of a tumor. Although the limitations of an organic sensitizer compromising the efficiency of the therapy are low water solubility and poor tumor specificity causing poor retention. Therefore, the combined effect of chemotherapeutic agent and sonosensitizer is required to overcome these deficiencies and to improve the biocompatibility of a nanosystem. A self-assembled nanosonosensitizer Ce6-PTX@IR783 with Ce6 as a hydrophobic organic dye, IR783 as hydrophilic organic dye attached to anticancer agent paclitaxel has been developed [35]. The hydrophobic nanomedicine showed good stability in an aqueous solution, high drug entrapment efficiency, and excellent monodispersity. The in vivo and in vitro tests showed synergistic photoacoustic imaging and Chemo-Sonodynamic anticancer therapy. Here, chlorine e6 (ce6) is a fluorescent dye that is safe and effective sonosensitizer in SDT. However, the dye has many limitations, such as, poor solubility, which makes it prone to aggregate in an aqueous solution. Paclitaxel (PTX) is an anti-tumor drug of broad-spectrum extensively used in the clinic [60]. Moreover, it has some defects such as, poor bioavailability, high hydrophobicity, and inadequate tumor penetration, which decrease its therapeutic effect. IR783 is a hydrophilic heptamethine cyanine dye which is promising as it is known for tumor-targeting capability and low toxicity. It shows delayed retention and specific accumulation in the tumor cells. As IR783 has the ability of fluorescence-imaging, it can be used for photoacoustic imaging (PAI) which combines the advantages of optical imaging with high resolution and high penetration by ultrasound imaging. Ce6-PTX@IR783 is a nanodrug that overcoming the obstacles of low bioavailability and poor solubility of hydrophobic components, Ce6 and PTX. The drug can efficiently accumulate at the tumor site owing to the desirable size (~70 nm).

#### 2.1.7. MnS-BSA for Breast Cancer Treatment with Combination Therapy

Manganese Sulfide (γ-MnS) being metastable can release Mn^2+^ ions upon degradation in an acidic environment. Hence, it is a promising material for pH-responsive MRI of cancer [61]. BSA is used to regulate the size of MnS nanoparticles by tuning the ratio of BSA and Mn^2+^. Chemodynamic therapy (CDT) kills tumor cells by producing hydroxyl ions from H_2_O_2_ at tumor sites [62]. It has been reported that Mn^2+^ can be released by the MnO_2_ shell on decomposition in the tumor microenvironment. This process requires GSH and it improves CDT efficiency. Therefore, γ-MnS nanoparticles can be used to treat cancer by CDT. H_2_S gas is a concentration-dependent bio-signaling molecule that can be metabolized in the mitochondria at nanomolar concentrations. The anticancer effect can be enhanced by combining CDT and H_2_S gas. H_2_S gas being a signaling molecule can cause vasodilation and decrease vascular tension in solid cancer tumors. Due to these properties, MnS@BSA (γ-phase manganese sulfide with bovine serum albumin, BSA as a biological template) as a biodegradable and size-controlled nanomaterial was synthesized for gas therapy primed CDT and MR [23]. The nanomaterial responds in a slightly acidic condition to release Mn^2+^ ions which can give a Fenton-like reaction and generate hydroxyl free radical in the presence of H_2_O_2_ of tumor cells. MnS@BSA can also generate hydrogen sulfide which can be used in gas therapy.

### 2.2. Lung Cancer

Lung cancer is the second most common malignancy of both men and women and the primary cause of death worldwide. Because of detection in an advanced stage, it is related to high mortality. More than one million people are diagnosed with lung cancer every year in India [63]. Lung cancer is mainly of two types, non-small cell (NSCLC) and small cell lung cancer, where NSCLC is the most common type of cancer, accounting for almost 85% of cases [64]. Some common causes of lung cancer are smoking, exposure to asbestos, air pollution, and prior history of cancer [65]. Based on the type of malignancy and stage, the lung cancer treatment comprises of chemotherapy, radiation therapy, and targeted therapy or combinatorial approaches [66]. Platinum-based drug treatments are first-line chemotherapy regimens in lung cancer. However, chemotherapy with platinum-based drugs is associated with dose-limiting side effects such as nephrotoxicity, cardiotoxicity, nausea, fatigue, etc. Therefore, to mitigate the effects, platinum-based drugs were used in combination with other anticancer agents for enhanced therapeutic efficacy. The unmet medical need demands newer strategies focusing on targeted toxicity to tumor cells only. Nanotechnology is an innovative approach that has recently gained significant clinical importance in cancer management. In this regard, the implications of some of the important nanomaterials used in lung cancer diagnosis and therapy have been discussed here.

#### 2.2.1. Anti-EGNO1 Tagged Gold Nanoparticles as Immunosensor for Diagnosis of Lung Cancer

Enolase (ENO) enzyme converts 2-phosphoglycerate into phosphoenolpyruvate during glycolysis. There are three isoforms of enolase present in mammalian cells: α-enolase (ENO1), γ-enolase (ENO2), and β-enolase (ENO3). It can be indicated that a high concentration of cerebrospinal fluid is correlated to astrocytoma. Patients expressing the highest level of cerebrospinal fluid ENO have the highest tumor growth rate [67]. Further, for small cell lung cancer (SCLC), elevated levels of ENO1and ENO2 can be a useful tumor marker [68]. Immunosensor is used as a biosensing device that depends on the reactions of antibodies to their corresponding antigens by quantifying a specific activity such as, fluorescence, bioluminescence, electrochemical signals, or radioactivity. A novel electrochemical immunosensor was synthesized by Ho et al. [69] featuring anti-EGNO1 tagged gold nanoparticle used as a bioprobe for the detection of trace ENO1 at picogram per ml level. Gold nanoparticles in conjugation with disposable screen-printed carbon electrodes (SPCE) make a versatile material for developing an electrochemical sensor. The sensor was fabricated by the physical absorption of PEG into SPCE. The probes for signal amplification were prepared by mixing anti-ENO1 and gold nanoparticles of 33 nm in diameter. The biosensor fabricated had a short response time, high sensitivity, and did not require sophisticated instrumentation.

#### 2.2.2. Gold Nanospheres and Peptide Conjugate as a Tool for Diagnosis of Lung Cancer

Multiplexing diagnostic platforms have unique advantages as it has higher throughput screening capacity, better working efficacy, and lower cost [70]. For biosensing, fluorescence and SERS techniques are best suited because they have a wide range of analyte detection and show high specificity and sensitivity. Fluorescence assays are being given attention as they can analyze complex biological events with high resolution. SERS is another promising diagnostic tool because of its high spectral specificity and excellent contrast [71]. Due to slow imaging speed, SERS has a low temporal resolution, which slows down the recognition of various target biomarkers within a limited time. Therefore, combination therapy of these techniques overcomes the disadvantages that have been created by their independent use. Saranya et al. [72] fabricated nanoparticles that were switchable between fluorescence and SERS. This nanosystem was made of gold nanospheres to the surface of which Raman active fluorophores were attached by positioning a peptide strategically. The enzymatic action of cathepsin B (cathB) is used to engineer cleavage of the peptide linker in the acidic microenvironment of a tumor. This cleavage facilitates the activation of fluorophores, which further increases the distance between the dye and gold nanoparticle surface. This caused the suppression of chemical enhancement and hindered SERS activity induced by the metal substrate. Detection of protein targets was done after it with monoclonal antibody by SERS encoded nanoparticle probes. This bimodal approach turned out to be highly efficient and was able to achieve recognition of single as well as multiple biomarkers that were in a complex biological environment by spectral tracking guided through multi-color tagging.

#### 2.2.3. Icotinib and DOX Co-Encapsulated in EDS Nanoparticle for Lung Cancer Treatment

Epidermal growth factor receptor (EGFR) is overexpressed in most patients having NSCLC and is a crucial target for therapy [73]. Although various chemotherapeutic agents as EGFR inhibitors have been created, they eventually showed drug resistance to EGFR inhibition. Therefore, it was concluded that NSCLC treatment was not successful by single-drug chemotherapy. Whereas, using combination chemotherapy could benefit cancer as combining different therapeutic modalities could target multiple mechanisms and pathways [74]. Li et al. [75] optimized the combination of DOX with three different EGFR inhibitors (erlotinib, apatinib, and icotinib) for the treatment for A549, NCI-H1975, and PC9 cancer cells. It has been previously reported that erlotinib and DOX show a synergistic effect in several breast cancer cell lines [76]. Apatinib with DOX has shown a synergistic effect in soft tissue sarcomas. For the NSCLC patients, it was reported that icotinib combined with chemotherapeutic agents, could improve the chances of survival. Icotinib in combination with DOX was found to be the optimal synergistic drug, which was co-encapsulated by hyaluronic acid and cationic amphipathic starch to form EDS nanoparticles. In vivo and in vitro experiments showed that targeted delivery was very effective due to EDS nanoparticles, and the accumulation of the drug in the tumor cell was enhanced. In vivo toxicity was decreased due to the reduction of nonspecific accumulation in healthy tissues. The synergistic effect of DOX and icotinib was successfully enhanced to show inhibition of NSCLC.

#### 2.2.4. Paclitaxel-Loaded Aerosol Nanoparticles for Drug Delivery in Lung Cancer

Intravenous drug delivery has been used extensively for treating lung cancer. However, such a systematic delivery system often has disadvantages such as low specificity, high toxicity, and high dosage. Therefore, the pulmonary delivery system seems to be a good alternative route for drug delivery for lung cancer as it can overcome the limitations related to intravenous delivery. One such technique is aerosol therapy that shows fewer side effects and lower doses and involves a non-invasive delivery system. The most preferred aerosol device is dry powder inhalers (DPI) as they are portable and easy to use, have stability and solid formulation, and a wide variety of ingredients and dosage can be delivered [77]. Micron sized particles are easily delivered and removed from the body by phagocytosis. These micro-sized particles are aggregates of nanoparticles, and when delivered deep in the airways, they dissociate back into nanoparticles, which can be easily deposited in the lungs. However, the advantages of using nanocarriers are that they possess the ability to penetrate physiological barriers, improve the stability of colloidal hydrophobic drugs, and can be used to reduce dosing frequency. The overall performance of the aerosol depends on its aerodynamic diameter, which means that larger particles deposit due to sedimentation, whereas smaller particles deposit by diffusion. Paclitaxel (PTX) is a chemotherapeutic drug that requires excipients for intravenous delivery as it has low water solubility and high toxicity [78]. Nanocarriers have been developed for PTX, as the hydrophobicity of PTX reduces its bioavailability. Recently, paclitaxel-loaded aerosol nanoparticles were synthesized and delivered to the lungs through a dry powder inhaler for treating NCSLC [79]. The size and morphology of the nanoparticles made them accessible to the distal region of the lungs.

#### 2.2.5. Protein-Decorated PLGA Biomimetic Nanocomposites for Drug Delivery in Lung Cancer

Drug delivery using nanoparticles has been often used because they tend to stay in systematic circulation for a prolonged period, achieve controlled release, and good tumor penetrating ability [80]. However, most of the nano delivery systems have some limitations in clinical trials. They show poor circulation or accumulation in the tumor site or poor penetration. This is due to the microenvironment of the tumor which might provide physical or biochemical barriers to nanodrugs. To overcome these limitations, nanoparticles with surface ligands such as, antibodies, enzymes, and folic acid were designed, which improved tumor targeting. Therefore, biomimetic nanoengineering seems to be a favorable strategy where RBC, platelets, or leukocytes like cell membranes can modify the nanoparticle surface [81]. H1975 membrane protein-decorated PLGA biomimetic nanocomposites were synthesized to subdue the drug resistance in EGFR-NSCLC cell lines [82]. This nanocarrier is made for chemoresistant NSCLS to enhance the therapeutic efficacy of DOX and icotinib. In the in vivo experiments, it was observed that on intravenous injection, the tumor inhibition rate was found to be highest, and there were minimum side effects.

#### 2.2.6. Bismuth-PEG Based Nanocarrier for Combination Therapy in Lung Cancer

Mesoporous materials can be used to decorate the outer layer of therapeutic agents [83]. The combination of chemotherapy and other therapeutic techniques can be carried out by the construction of this core-shell nanostructure for tumor inhibition. Metal porous materials and metal-organic frameworks are other such nanostructures that can have a therapeutic function and can load cancer drugs for synergistic oncotherapy [84]. According to recent studies, Bismuth (Bi) has been known to be a green metal as it has a high atomic number (Z = 83), low melting point, high X-ray attenuation, nontoxicity, and is not radioactive. These properties of Bi have made it an important nanomaterial in different fields such as diagnosis and therapy of cancer. Some studies have shown that Bismuth-based nanomaterials such as its oxides, sulfides, and selenide are outstanding materials for radiotherapy enhancement performances and computed tomography contrast [85]. Core-shell heterojunctions can be used for building theranostic by combining these materials with other magnetic resonance enhancement nanomaterials (MnSe, MnS, and Fe_3_O_4_). To enhance the therapeutic effect and diagnostic efficiency, imaging reagents and the chemotherapeutic drug can be introduced to Bi-based nanomaterial [86]. Bismuth-based litchi shaped mesoporous nanoparticles were synthesized as a nanocarrier for DOX and as a radiosensitizer [87]. A mesoporous structured Bi-based nanomaterial with good loading capacity, low cost, large scale fast, and facile synthesis has not been reported previously. The in vivo study showed enhanced biocompatibility and water dispersibility on assembling the nanoparticles with PEG. The combination therapy was improved, which could be used as CT imaging-guided chemotherapy. A 20% Yb containing NBOF (Na_0.2_Bi_0.8_ O_0.35_F_1.91_) Bi-based mesoporous nanomaterial was used for drug load and controlled release. It was synthesized in 1 min, and DOX was loaded on it as it has a high loading capacity to develop a smart drug delivery system.

### 2.3. Skin Cancer

Exposure to the sun can cause abnormal cell growth and can lead to skin cancer. Basal cell carcinoma, melanoma, and squamous cell carcinoma are the three major types of skin cancers. Some of the skin cancer symptoms are large dark spots, a mole that changes its color and size, or bleeds. The exposure to ultraviolet radiation coming from the sunlight and the light in tanning beds have been known to cause DNA damage. Melanoma has a very high tendency to metastasize to other parts of the body, such as the lungs, lymph nodes, liver, heart, and brain [88]. This property of melanoma cancer makes it deadly as it lowers the survival time of patients. Non-melanoma skin cancer corresponds to 5.8% of all cancers globally. Primarily such cancer types could be removed by surgical interventions and radiation/chemotherapy. However, the physical and emotional impact suffered by patients remains a major challenge as most tumors appear in sun-exposed areas. Treatment of skin cancers has improved over several decades, but the survival rate of patients with an advanced stage is low. The nanotheranostics focuses mainly on combined imaging and therapeutic strategies for localized tumor treatment while improving drug penetration at the tumor site. In the light of nanotheranostics, some of the important nanomaterials used in the treatment of skin cancer detection and therapeutics are discussed below.

#### 2.3.1. Indium Nitride Nanoparticles Used for the Detection of Skin Cancer

During the past decades, various studies have been to develop non-invasive and highly sensitive diagnostic techniques for skin cancer [89]. A few such techniques have been developed previously using the optical mechanism to differentiate between the cancerous and healthy tissue. Terahertz (THz) wavelengths are such examples because of less scattering and longer penetration depth (0.5 to 6.5 mm) than visible wavelengths. THz is sensitive to water content in the local tumor area, shaping the reflected electromagnetic field (EMF). A contrast-based image can be procured by this method between the healthy and unhealthy tissues by calculating the change in intensity of reflected field as it has been established that skin cancer causes an elevation in the water content level [90]. However, THz wavelengths are longer than near-infrared (NIR) and visible (VIS), which causes its imaging to be of poor resolution. Though recently, many studies have been done to improve imaging techniques. The results have been very fruitful because the resolution enhanced to several hundred nanometers sufficient for the detection of early skin cancer [91,92]. Similarly, another sensing technique is localized surface plasmon resonance (LSPR), in which gold or silver nanoparticles are used for biomedical sensing at visible and near-visible wavelengths [43]. The advantage of this technique is higher sensitivity to local changes in the tissue due to the enhancement of EMF around the nanoparticles. Polarized light imaging is another technique developed in NIR and VIS frequencies. It provides better contrast imaging and higher sensitivity in comparison to nonpolarized imaging. This method creates an artificial image with better contrast between normal and abnormal tissue instead of other imaging techniques with intensity images of low resolutions. A method to improve the cancer detection technique by combining the advantages of LSPR and polarized light imaging with THz imaging was proposed [93]. It was achieved using spherical nanoparticles of indium nitride InN, a material presenting LSPR at the THz domain. Parylene-C coated InN demonstrated both biocompatibility and high sensitivity to water content.

#### 2.3.2. Nanostars Coated with RBC and Platelet Membrane for Drug Delivery in Skin Cancer

**Figure 3:**
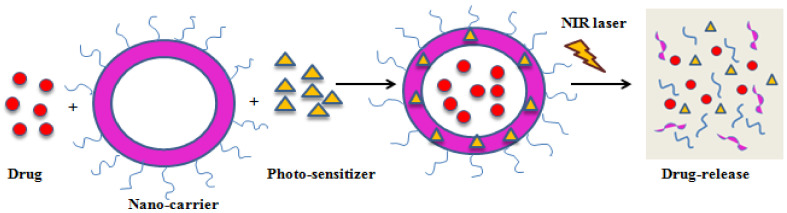
Schematic illustration of PDT-mediated drug release.

Hydrophobic drugs can be successfully loaded onto phospholipid bilayer without any conjugation in membrane coated biomimetic drug delivery systems. Therefore, it is evident that the membrane coating strategy improves the biocompatibility of the nanoparticles and their bioavailability [94]. PTT is a non-conventional cancer treatment strategy, safer and non-invasive than other techniques because of higher accuracy [95]. Gold nanoparticles are the most promising PTT agents as they have high adjustable properties such as, particle size, shape uniformity, and surface modification, which improve their treatment efficacy [96]. PEG is usually used to enhance the stability, solubility, and immune escape of these gold nanoparticles. Although PEGylation has its own disadvantages, on repetitive administration and in the presence of anti-PEG bodies, therapeutic efficacy is reduced [97]. Whereas, red blood cell membranes have a better immune escape ability because the proteins present in them act like immunomodulatory antigens to escape phagocytosis. PTT kills tumor cells by generating high temperatures, but the exact mechanism is unclear. It is known to cause cell death at 42 °C, which can be very harmful to the healthy cells around the tumor. To overcome this disadvantage, PTT could be combined with other anticancer therapies to achieve better therapeutic outcomes. PTT combined with chemotherapy can be a much safer combination therapy to improve therapeutic efficacy under lower temperatures. As cell membranes are sensitive to temperature change; they can allow the release of chemotherapeutics by change in temperature. This property helps in preventing damage to healthy cells and maximizes the therapeutic efficacy of the nanoparticles (Figure 3) [98].

Active targeting by gold nanoparticles without any functionalization is impossible. To overcome this, the platelet membrane can be used for direct targeting. Platelets can recognize the cancer cells easily and attach themselves to the tumor, causing maximum damage to tumor cells. Based on these factors, gold nanostars with curcumin (R/P-cGNS) were synthesized and coated with RBC and platelet membrane [20]. It was noted that the RBC membrane provided antigens, whereas platelet coating improved the targetability of cancer cells. The results showed that R/P-cGNS enhanced anticancer effects and can deliver drugs as well as avoid macrophage phagocytosis.

#### 2.3.3. QW-296 Polymeric Nanoparticle for Drug Delivery in Skin Cancer

The efficiency of cancer therapy drugs mainly depends on toxicity caused by off-targeting and mechanisms of chemoresistance [99]. Since melanoma aggressively metastasizes, addressing the following issues can be beneficial for developing an effective drug for the treatment of cell migration and invasion, therapeutic resistance mechanism, and off-target dosage [100]. Indeed, therapeutic options are limited for metastatic melanoma, tubulin inhibitor having high efficiency and low clinical resistance because binding sites of colchicine can inhibit the rapid division and metastasizing of cells of tubulin protein.

The nanoparticle-based delivery platform enhances the retention and permeability of the cancer drugs and the potential to deliver drugs to the target site with low toxicity to healthy tissues around [101]. The pharmacokinetic profile of a drug is enhanced by using nanoparticles that improve the solubility of a hydrophobic drug, bioavailability, and circulation time. Micellar delivery was applied to enhance the aqueous solubility of 4-substituted methoxybenzoyl-aryl-thiazole-100 (SMART-100) and bicalutamide with methoxy poly (ethylene glycol)-b-poly (D, L-lactide) (mPEG-PLA) polymer [102]. The micelles prepared had low drug loading ability, requiring a new, safer, and more biocompatible polymer. Recently, a novel tubulin destabilizing agent named QW-296 encapsulated in polymeric nanoparticles was reported [103]. A new analog was synthesized and conjugated with methoxy poly (ethylene glycol)-b poly (2-methyl-2-carboxyl-propylene carbonate-g-dodecanol) (mPEG-bPCC-g-DC). This improved the payload of drugs and prevented premature drug release. In the melanoma mouse model, polymeric micelles with 14.3% drug payload showed good inhibition to tumor growth. It was observed that QW-296 significantly inhibited metastatic melanoma cells’ cell proliferation, leading to cell apoptosis and cell death.

#### 2.3.4. Amino acid Modified Gold Nanoparticle for the Treatment of Skin Cancer by PTT

Gold nanoparticles have been developed with targeting moieties like antibodies and peptides to avoid off-targeting and to achieve tumor-specific therapeutics [104]. This approach enhances tumor selectivity. However, its practical application is still a challenge because of the high cost, quick clearance from the immune system, and instability of proteins in the biological system [105]. It has been noted previously that only very few nanoparticles modified with targeting proteins could reach the actual tumor site resulting in poor selectivity. Off-targeting can be very harmful to healthy tissues. To overcome this issue, in situ assembly of small gold nanoparticles as photothermal agents may provide selective therapy and would cause less damage to the healthy tissues and organs. Aggregation based light-dependent gold nanoparticles have been prepared by dimerization molecules using UV or visible light. However, because of the low penetration ability of such light due to absorption by body fluids, hemoglobin, and other tissue, it becomes a challenge to do in vivo experiments of these photothermal therapies [106]. In a recent study, pH-responsive gold nanoparticles have been synthesized that can accumulate around the tumor with better selectivity in PTT [107]. Although, non-selective tissues could have similar microenvironments with the same pH causing accumulation of nanoparticles and unfavorable consequences. Hence, there has to be a development of a much more sensitive method to accurately assemble these nanoparticles for a much more effective PTT.

Amino acid-modified gold nanoparticles were synthesized and selectively assembled at the tumor site for PTT [108]. In this work, for the tumor therapy, catalyzed polymerization of amino acids on gold nanoparticles surface was done by the intracellular enzyme (transglutaminase, TGase) to achieve control over PTT. As TGase is abundantly present in tumor cells, to avoid any unwanted triggering of polymerization of normal cells, a pH-responsive zwitterion surface was developed. This surface of zwitterion can display resistance to endocytosis in healthy tissues, enhances cellular uptake. In this synergistic therapy, the Fenton reaction takes place and hydroxyl radical is produced which is known to cause cell damage. The nanodevice showed 8-fold cytotoxicity against cancer cells and complete inhibition after a few weeks.

#### 2.3.5. Au-NCNC as a Nano-Drug Carrier for Treating Skin Cancer

CNTs are ideal drug carrier as they can be surface functionalized easily. Nonetheless, due to the tumor being heterogeneous, the modified nanomaterials for tumor targeting in the immunosuppressive microenvironment has been a difficult task. In recent studies, myeloid-derived suppressor cells (MDSC) being heterogenous have played an important role in regulating tumor progression and anti-tumor immune response [109]. It is known that MDSCs are produced in the bone marrow and spreads to the body through the bloodstream, and finally gets accumulated in the lymphoid and tumor. There are two populations of tumor-bearing MDSC hosts: polymorphonuclear cells (PMN-MDSC) and monocytic cells (MMDSC), which can promote metastasis, tumor cell invasion, and angiogenesis due to their immune suppressive activity [110].

A generally accepted approach is to target MDSC with a chemotherapeutic agent such as cisplatin or gemcitabine to induce cell death [111]. Chemotherapeutic agents at high concentrations have known to be highly toxic and may cause neuropathy or hypersensitive reactions. In previous studies, it has been noted that a low dosage of paclitaxel can regulate the accumulation and function of MDSC in melanoma and increase immune-stimulatory. However, the actual application of these drugs is limited due to the immunosuppression, non-specific effects, cumulative toxicity, and metastatic activity. Therefore, there is a need to develop a novel approach to controlled drug delivery.

The synthesis of nitrogen-doped carbon nanotube cups (NCNC) corked with gold nanoparticles as a nano-drug carrier with loaded paclitaxel was reported [112]. Administering this to the tumor generates reactive nitrogen and oxygen species by inhibiting myeloid-derived suppressor cells, which cause inhibition of cancer cell growth. The immunosuppressive activity of cells was diminished when Au-NCNC was loaded with paclitaxel and delivered to activate MDSC. Since Au-NCNC is 200 nm long, could not penetrate the tumor microenvironment according to the enhanced permeability and retention effect. However, MDSC has overexpressed oxidative biodegradation, due to which it is expected that AU-CNCN would degrade selectively in circulating and lymphoid tissue [112]. This would result in MDSC differentiation and the local delivery of paclitaxel. It was reported that melanoma growth in mice was inhibited by 25–30%, and the mice were completely cured in 2–3 weeks after treatment with Au-NCNC loaded paclitaxel. On tracking gold nanoparticles, it was observed that Au-NCNC was localized in the tumor and was also found in the spleen and liver, which caused a decrease of MDSC as confirmed by flow cytometry. It was further proved that targeting of MDSC showed antitumor effects even without specific targeting in the tumor microenvironment.

### 2.4. Prostate Cancer

Prostate cancer in man is one of the leading causes of mortality worldwide (3.8% deaths globally). Men above the age of 55 with a family history are prone to get diagnosed with prostate cancer. Prostate cancer has various symptoms such as poor bladder control, blood in the urine, loss of appetite, and weight [113]. Some of the therapies for the treatment of prostate cancer are radiation therapy, cryotherapy, hormonal therapy, chemotherapy, and immunotherapy [114]. Despite several advancements in conventional therapies, there is an urgent requirement for specific treatment modalities for prostate cancer management. Nanotechnology is rapidly gaining attention in the field of medicine, as various nanoparticles showed clinical efficacy in cancer management. Some recently developed nanomaterials with surface modifications used in prostate cancer therapy have been discussed here.

#### 2.4.1. Gd@Cy5.5@SiO2 Nanoparticles for Theranostics of Prostate Cancer

Diagnosis of prostate cancer can be made by MRI as it possesses high soft-tissue resolution. However, MRI cannot diagnose prostate cancer at an early stage, neither can it differentiate from chronic prostatitis [115]. After initial treatment, especially by radiation therapy or surgery, it is common to have a biochemical reoccurrence. In such cases, it becomes challenging to identify metastases or tumor reoccurrence by convention imaging techniques as they have poor specificity and low sensitivity [116]. Compared to conventional imaging techniques, a combination of multiple molecular imaging techniques will be able to achieve better results due to their combined advantages. In recent studies, multimodal probing techniques showed incredible new possibilities in the field of cancer diagnosis [117]. One such technique that has attracted attention is MRI/fluorescence bimodal imaging probe. Fluorescence has good sensitivity, but it lacks spatial resolution, whereas MRI has multidirectional and multiparametric imaging properties, which improve its structure and provides much more detailed information. Therefore, it is an excellent bimodal imaging technique for the diagnosis of tumor cells.

Nanomaterials, as carriers for bimodal imaging, have become a common technique for improved results. CNTs, silica nanoparticles, liposomes, and polymeric micelles are some of the carrier materials commonly used. The sol-gel method is used to synthesize silica nanoparticles. Since silica nanoparticles have high colloidal stability, biocompatibility, adjustable particle size, transparency to light, no magnetic field, and low toxicity, it has become a promising material for biomedical application and is, therefore, has witnessed explosive growth over time [118]. Other than that, silica also has hydroxyl groups, which allow modification with different chemical moieties for drug and antibody loading [119]. Gadolinium-doped silica nanoparticles have shown a longitudinal relaxation rate. Gadolinium and fluorescent dye named Cy5.5 were used to achieve MRI/fluorescence bimodal imaging [31]. The dye in the NIR region has low autofluorescence interference and high imaging sensitivity, which enables accurate positioning of the probe. However, it is important to avoid non-specific interaction between healthy cells and particles in target cell imaging. Therefore, to avoid the accumulation of proteins in the blood serum that lead to endocytosis and improve the circulation time of nanoparticles, PEG has been used.

Gd@Cy5.5@SiO2 nanoparticles were prepared in conjugation with the antibody YPSMA-1 against prostate-specific membrane antigen (PSMA) to improve the target specificity of prostate cancer imaging material [120]. PSMA is a transmembrane protein of type II that is specifically present in prostate cancer cells with high uniformity and frequency and some other normal tissues. Therefore, it is an ideal targeting agent for the imaging and treatment of prostate cancer. PEG-coated and Gd-loaded targeted fluorescent silica nanoparticles were synthesized by reverse microemulsion and carbodiimide method [120]. The biosafety and targeting ability of the nanoparticles was confirmed by in vivo and in vitro studies. The nanoparticles were highly stable, and approximately 1% of Gd^3+^ ions were found to be released only after 120 h.

#### 2.4.2. IGC and Ce6 Encapsulated in HSA for Drug Delivery in Prostate Cancer

For the treatment of prostate cancer, PDT has been a newly emerging strategy [121]. In PDT, photosensitizer molecules are irradiated with a laser of the appropriate wavelength. These molecules further transfer their energy to molecular oxygen in the surroundings to convert them into singlet oxygen (^1^O_2_) to kill cancer cells. Although, there are a few disadvantages of using PDT as they can accumulate in tissues of the patients, especially in eyes and skin. These can be activated in sunlight, which can cause various side effects like skin photosensitivity. A second-generation photosensitizer, chlorine e6 (Ce6) has a higher production rate of singlet oxygen, and it is known to have efficient phototoxicity on tumor cells [122]. However, Ce6 is always actively phototoxic to the normal tissues, which limits its clinical use. To overcome this drawback, activatable PDT was designed. This could control the photosensitizer’s activity and turn it off for the normal tissues while turning it on for cancer cells. The photosensitizer is developed such that its photosensitivity can be switched off before administration, and nanoparticles such as gold and graphene oxide, pH, reactive oxygen species, and enzymes can be used to trigger the turn “ON” of the photosensitizer [123]. In the case of gold nanoparticles, they exhibit strong surface plasmon resonance in the NIR region can act as a quencher or photosensitivity, causing suppression of the production of singlet oxygen by gold nanoparticles. However, most of the materials used in PDT are non-biodegradable, which has limited its clinical application [124].

Indocyanine green dye as a quencher was used for Ce6 photosensitizer as it a safe cyanine dye widely used for clinical purposes [125]. Both ICG and Ce6 were encapsulated in human serum albumin (HSA) nanoparticles and were highly biocompatible. As the absorbance band of ICG overlaps with the emission spectrum of Ce6, therefore ICG can quench the phototoxicity of Ce6. Upon irradiation at 880 nm, the ICG degrades, which causes the recovery of Ce6’s photosensitivity, whereas above 660 nm Ce6 is activated and generates singlet oxygen for PDT.

#### 2.4.3. Glyconanoparticles for Targeted Delivery in Prostate Cancer

Multivalent carbohydrate-binding proteins are attractive molecular targets as they mediate dangerous cellular activities. By interaction between immune cells, endothelial, stromal, and tumor, Galectin-1 (Gal-1), which is a homodimeric β-galactoside binding lectin, causes tumor progression [126]. Gal-1 has various intracellular and extracellular functions and is present both inside and outside the cells. Gal-1 is involved in the binding of glycoproteins, cross-linking of receptors, and forming large aggregates. Gal-1 is upregulated during prostate cancer progression and is a potential target for drug delivery to reduce cell proliferation and survival of cancer cells by using β-galactoside functionalized-nanoparticles.

To enhance the affinity of glycol-conjugates, glycopolymers, glycopeptides, and glycodendrimers could be functionalized with gold, iron oxide, silica nanoparticles. Several factors improve the binding and targeting capability of glyconanoparticles, such as the arrangement of carbohydrate ligands on the surface of nanoparticles, ligand density, and the hydration state of the binding site. For the nano delivery vehicle to successfully target the cancer tissue, it should have a long circulation lifetime, the release of the drug should be easily done by cellular internalization stimuli, and the final degraded product should not be toxic. Therefore, surface properties of glyconanoparticles need to be enhanced by controlling toxicity, colloidal stability, hydrophilicity, drug loading capacity, degradability, and immunogenicity. To avoid aggregation and interaction with other serum proteins, the conjugation chemistry of the nanoparticle should be carefully designed so it should be biocompatible and biodegradable.

Glycogen nanoparticles were used to design a multivalent glyconanoparticle with a high affinity for prostate cancer [127]. Glycogen nanoparticles are highly branched found in mammals, play an important role in osmotic pressure regulation and blood glucose homeostasis. They are composed of molecular spheres (β-particles), and larger aggregated rosettes of smaller particles (α-particles), and the spherical glycogen particles are 20-150 nm in diameter, having a hydrophilic surface with the non-reducible end of glucose. Glycogen is highly water-soluble, biocompatible, biodegradable, and available in abundance. In this case, to functionalize glycogen nanoparticles, copper(I)-catalyzed alkyne-azide cycloaddition chemistry was used. The lactose moieties used for this purpose provided β-galactoside moieties at the terminal to improve target interaction on prostate cancer cells.

#### 2.4.4. Cu (DDC)_2_ Nanoparticles as High Concentration Drug for Prostate Cancer Treatment

To treat an early stage of prostate cancer is relatively easy than the treatment at a later stage with aggressive and metastatic cancer cells. At the later stage, prostate cancer migrates outside the prostate gland and even starts to resist hormone therapy, which is usually treated by chemotherapy with taxanes [128]. However, taxanes did not get good responses from patients, and drug resistance to prostate cancer in various cases was developed. Recently, alternate strategies have been given attention, such as drug repurposing or repositioning. Disulfiram (DSF) has been used for treating alcohol addiction for many years, but recently, it has been identified as an anti-cancer agent and shows excellent properties in combining with copper ions [129]. DSF/Cu can sensitize resistant cancer cells to chemotherapy agents by inhibiting P-glycoproteins and stem cells of cancer. The formation of copper diethyldithiocarbamate Cu(DDC)_2_, which is an active metabolite, controls the anticancer efficacy of DSF/Cu. However, a very low concentration of Cu(DDC)_2_ would be administered in vivo due to the rapid degradation of DSF and poor solubility compromising the drug efficacy [130]. Hence, a more effective approach was needed to improve the concentration of drugs being administered to achieve an anticancer effect. In a previous study, the injectable form of Cu(DDC)_2_ has been developed by using Metaplex technology, which uses liposomes to synthesize Cu(DDC)_2_ from diethyldithiocarbamate (DDC^−^) present in the core of liposomes and copper ions by acting as a nanoscale vessel. The nanoparticles were then incorporated in liposomes and stabilized by liposome membranes, although the process becomes highly expensive for large-scale production.

A novel stabilized metal ion ligand complex (SMILE) technology to synthesize Cu(DDC)_2_ nanoparticles were designed [131]. This technology has a simple formulation and is quite a cost-effective process. These advantages make it favorable for mass production at reasonable pricing. The high concentration of the drug, controlled release properties, and good low efficiency can be achieved on the preparation of Cu(DDC)_2_ nanoparticles with this technology.

#### 2.4.5. Docetaxel-Tannic Acid Self-Assembly as an Anti-Cancer Drug for Prostate Cancer

Due to chemotherapy stress, an irreversible growth arrest like state arises, which is known as cellular senescence. It is characterized by the secretion of cytokines, proteases, chemokines, and shortening of telomeres. As senescence regulates cell proliferation rates of cancer cells, its accumulation could cause chemoresistance or cancer relapse [132]. Therefore, to eliminate cells expressing the senescence-associated secretory phenotype (SASP), an advance therapeutic strategy is needed. A plant-derived polyphenol named Tannic acid (TA) shows anticancer effect against many types of cancers and prevents chemoresistance against cancer cells [133]. However, it is required in high concentration to be effective as an anticancer drug. Therefore, it cannot be used alone as an anticancer agent. Whereas Docetaxel (Dtxl) has been delivered by nanoparticles previously, although it failed to prevent chemoresistance and senescence phenotype in cells. To overcome these limitations, Tannic acid-based Dtxl by self-assembly techniques for treating prostate cancer were synthesized [134]. Through hydrogen and ionic bonding, TA could form self-assemblies and solubilize Dtxl. The docetaxel-tannic acid self-assemblies is an efficient anti-cancer drug as it successfully transports Dtxl to xenograft tumor and prostate cancer cells and blocked cellular senescence.

## 3. Conclusions and Prospects

Nanodrugs have received a tremendous amount of attention from researchers as a tool for cancer therapy. Due to some advantages of nanomaterials, they have been increasingly used in photothermal, photodynamic, chemo, and radiation therapy to enhance the therapeutic effect of the nanodrug. Nanomaterials that exhibit good cellular uptake, prolonged circulation time, and site-specificity are being explored for imaging and diagnosis of cancer, and even as a drug carrier for targeted drug delivery. Moreover, having a high surface to volume ratio, nanomaterials can load numerous drugs that can be delivered to the target by enhanced permeability and retention effect. Hence, nanomaterial-based therapeutics show superior anti-cancer efficacy while reducing the side effects. In this review, various nanomaterials have been discussed, such as gold nanoparticles, quantum dots, polymer nanoparticles, carbon nanotubes, and polymer-drug conjugates for breast, lung, skin, and prostate cancer. To improve the biocompatibility of the nanoparticles, they are conjugated with different biomaterials and membranes. These modifications provide combination therapy, which can overcome drug resistance, commonly observed in cancer. These studies have incredible potential to develop the nano-medicinal field of clinical cancer therapy. Some of the novel nanomaterials have been accepted for cancer treatment commercially.

Despite all these advantages, it has been a challenge to successfully translate the properties of nanomaterial-based therapeutics into clinical trials. Several concerns remain including in vivo targeting efficiency and pharmacokinetics. Most importantly, little has been known about the toxicity of nanoparticles and the health risk they pose to the human body. The clinically approved nanodrugs possess biocompatibility achieved only by modification of nanomaterials with membranes or other biomaterials. The metal nanoparticles, quantum dots, and other nanomaterials are nonbiodegradable, which enhances cytotoxicity as they retain inside the system long after administration. Therefore, for better clinical value, the nanomaterials need to be biodegradable and biocompatible. Despite these limitations, nanomaterial-based cancer therapy has provided a direction for future development in the field of diagnosis, imaging, and combination therapy of cancer. Shortly, it is expected that many such drugs will be designed and approved by clinical trials.

## Figures and Tables

**Table 1:** Nanomaterials used in cancer diagnosis and therapy.

S. No.	Type	Features	Function	Cancer	Observation	Validation Level	Ref.
1.	Metal NP	Platelet-like membrane coated Au-Nanostars containing curcumin	Controlled release under NIR irradiation to target melanoma cells and to have an immunomodulatory effect on macrophages. Nutraceutical curcumin shows anti-cancer and anti-inflammatory effects.	Skin cancer	B16-BL6 melanoma cells	In vivo cytotoxicity by MTT assay Immune responses in the animal model	[20]
2.	Metal-based peptide	Glutathione and iron redox couple	Novel glutathione (GSH) and iron redox couple sequentially triggered LPO generator, which supplied the Fenton reaction-independent downstream executioner of ferroptosis for cancer therapy.	Breast cancer	Ferroptosis therapy	MTT assay Mouse breast cancer 4T1 cells for in vivo tests	[21]
3.	Metal/metal oxide NPs.	Copper/Copper oxide NPs	Cu/CuO NPs were cytotoxic and genotoxic to both normal and cancerous lung cells	Lung cancer	Gain easy entry into the body through the skin and the respiratory system.	In vitro study of carcinoma lung cells (A549)	[22]
4.	Manganese-based nanomaterial	MnS@BSA as a biological template	MnS@BSA can responsd in the mildly acidic microenvironment and release Mn^2+^ for Fenton-like reaction to generate •OH in the presence of endogenous H_2_O_2_ of tumor cells.	Breast cancer	Gas therapy primed hemodynamic therapy and MRI imaging	In vitro Combination Therapy In vivo Imaging and Biodistribution, Combination therapy	[23]
5.	Metal NPs.	Zinc oxide nanoparticles	Low concentrations of nZnO resulted in cell cycle arrest at S phase, facilitated cellular late apoptosis, repressed cell invasion and migration.	Urinary bladder carcinoma	Low dose exposure	Cell Apoptosis Detection and Cytotoxicity Assessment	[24]
6.	Gold-Nanobipyramid-Based Nanotheranostics	ICG-conjugated mesoporous silica-coated Au-nanobipyramid	Under the guidance of FL/PA imaging, GNB@SiO_2_-ICG exhibited remarkably enhanced therapeutic efficacy, which could eliminate the tumor tissues.	Skin cancer	Photothermal therapy	Cytotoxicity Assay In vivo FL/PA imaging, PTT	[25]
7.	2D metal boride	Monolayer Bi-anchored manganese boride nanosheets (MBBN)	A microwave-assisted chemical etching route was utilized to exfoliate the MBBN-constructed flower-like MBN, and a coordination-induced exfoliation strategy was further developed to separate the MBN into the dispersive monolayer MBBN.	Breast, kidney, Gastric cancer	NIR-photothermal and photoacoustic effects, MRI imaging properties	Cytotoxicity, Photothermal imaging In vivo (PTI), CT imaging, MRI, photoacoustic imaging, and tumor therapy	[26]
8.	Manganese dioxide nanomaterials	Glucose oxidase (GOx) armed manganese dioxide nanosheets	The as-prepared MNS-GOx can perform the circular reaction of glucose oxidation and H_2_O_2_ decomposition for enhanced starvation therapy. The hyperthermia of MNS-GOx could further improve the catalytic activity of GOx upon near-infrared laser irradiation.	Skin cancer	MR/PA dual-modal imaging-guided self-oxygenation/hyperthermia dually enhanced starvation cancer therapy.	In vitro and in vivo Synergistic Therapy In vivo MR/PA Dual-Modal Imaging	[27]
9.	Metal oxide NPs	SRF(sorafenib)@MPDA (mesoporous polydopamine)-SPIO (superparamagnetic iron oxide) nanoparticles	Sorafenib (SRF) and ultrasmall SPIO nanoparticles were loaded into the mesopores SRF@MPDA-SPIO nanoparticles. SPIO loading endowed the system with iron-supply for ferroptosis and made the system MRI-visible. SRF was able to induce ferroptosis in cancer cells.	Colon cancer	MRI and PTT	In vitro SRF and Fe release, MR imaging, Cellular uptake and cytotoxic by MTT assay In vivo biodistribution, MR and IR thermal imaging and cancer therapy	[28]
10.	Metal-organic framework	L-Cysteine decorated Zr-based metal-organic framework	To deliver cisplatin and HDAC inhibitor by PEG-modified biocompatible multifunctional CDDP-VPA@ZrMOF-Cys-PEG nanoparticles	Lung cancer	Chemotherapy combined with microwave thermal therapy	Immunofluorescence assay mouse normal fibroblasts and human lung adenocarcinoma A549 cells	[29]
11.	Functionalized Carbon Nanotubes (CNT)	Multi-walled CNT functionalized with magnetic Fe3O4 and Au-NPs	The combination of hyperthermia and radiotherapy, synergistically, caused a significant reduction in X-ray doses.	Breast cancer	Thermotherapy and radiotherapy Ultrasounds, CT scan, and MRI imaging	Viability assays	[30]
12.	Nucleic acid nanotubes	Aptamers conjugates straight and twisted DNA nanotubes	Aptamers functionalized nanomaterials enhance the targeting of nanomaterials and improve the stability of the aptamers.	Lymphoma	Chemotherapy and bioactivity investigation	Anticancer activity by CCK8 assay on K299 cells.	[31]
13.	Functionalized r-GO nanostructures	Lipid-Functionalized reduced Graphene Loaded manganese superoxide dismutase (hMnSOD)	The attachment of hMnSOD to lipid-rGO demonstrated multiple benefits for the lipid-functionalized graphene system due to its ability to impede cancer cell division without initiating necrosis and the lack of detrimental reactions with healthy breast cells.	Breast cancer	Photothermal properties and high loading capacity for cancer-fighting molecules	LIVE/DEAD assay for cytotoxicity MTS assay for cell proliferation	[32]
14.	Nucleic acid nanocarriers	Affibody-DNA tetrahedrons	The nano-structural drug contained one DNA tetrahedral core, an affibody molecule attached to one end of a polymeric FUdR oligonucleotides tail for targeting HER2.	Breast cancer	Targeted drug delivery to HER2-positive breast cancer	In vitro cytotoxicity In vivo antitumor study	[33]
15.	Lipid modified metal sulfide nanomaterial	Biomimetic lipid-modified WS_2_	Lipid coating strongly enhanced the stability of WS2 nanosheets on DOX loading, and WS2-lipid had a good photothermal performance and drug loading amount.	Breast cancer	Photothermal and chemo combination therapy	In vitro cytotoxicity In vivo Antitumor study	[34]
16.	Organic dye Nano sonosensitizer	Ce6-PTX@IR783, hydrophobic organic dye Ce6 hydrophilic organic dye IR783	Ce6 enhanced sonodynamic effect, while PTX exerted chemotherapeutic effect, and IR783 was applied to increase tumor-specific accumulation and assisted in fulfilling photoacoustic imaging.	Breast Cancer	Photoacoustic imaging and Chemo-Sonodynamic Breast Cancer Therapy	In vitro drug-releasing assay In vivo synergistic therapeutic effect	[35]
17.	Nanonzymes semiconductor biocatalyst	Fe_3_O_4_@Bi_2_S_3_ nanocatalysts (F-BS NCs)	Nanocatalysts caused irreversible damage to malignant cells but did not harm normal tissues strongly depended on the unique action of each component of composite nano-enzymes-semiconductor biocatalysts.	Solid tumor	Photothermal therapy, Infrared thermal and photoacoustic imaging	Cell Apoptosis and ROS detection. Photothermal Ablation and Chemotherapy Animal Tumor Model and Synergistic Phototherapy	[36]
18.	PEG decorated nanodrug	PEG decorated hydroxycamptothecin (HCP T) and bi-functional methotrexate	Owing to the pH-responsive property of PEG on the surface, the nanodrug exhibited excellent tumor targeting due to the prolongation of circulation time by PEGylation and the active targeting triggered by re-exposing MTX under acidic conditions.	Breast Liver, Kidney, Spleen, Lung, and Heart cancer	Chemotherapy and photoacoustic imaging	In vitro drug release, cellular uptake efficacy, pharmacokinetics, and biodistribution In vivo fluorescence and photoacoustic imaging	[37]
19.	Core/shell interface	Silver core/AIE (aggregation-induced emission) shell nanoparticles	Five imaging and therapy modalities (FL, CT, PA, PTT, and RT) were achieved with a single structural unit for sensitive tumor imaging and effective therapy.	Breast cancer	CT and radiation therapy, photothermal, and photoacoustic imaging.	Cell viability test and Flow cytometry assay Xenografted tumor models in vivo	[38]
20.	Quantum dots	Doxorubicin-loaded carbon quantum dots	Red-emissive carbon quantum dots can enter into the nuclei of not only cancer cells but also cancer stem cells.	Breast cancer	Chemotherapy	Cytotoxicity by CCK-8 assay In vivo imaging and biodistribution	[39]

## Data Availability

Not applicable.
